# Membranous desquamation of the hand in a 7‐year‐old boy with mild COVID‐19

**DOI:** 10.1002/ccr3.6651

**Published:** 2022-12-05

**Authors:** Masaki Tago, Risa Hirata

**Affiliations:** ^1^ Department of General Medicine Saga University Hospital Saga Japan

**Keywords:** COVID‐19, membranous desquamation

## Abstract

A 7‐year‐old Japanese boy was diagnosed with coronavirus disease 2019. He developed intermittent fever and headache, and the symptoms improved by Day 3. However, he developed membranous desquamation without erythema or swelling on the right hand on Day 4, which improved without treatment.

## Abstract 2

Because COVID‐19 can cause membranous desquamation regardless of severity, the general condition, vital signs, and other physical findings should be carefully confirmed in children to rule out complications of MIS‐C or Kawasaki disease.

## 1 CASE

**FIGURE 1:**
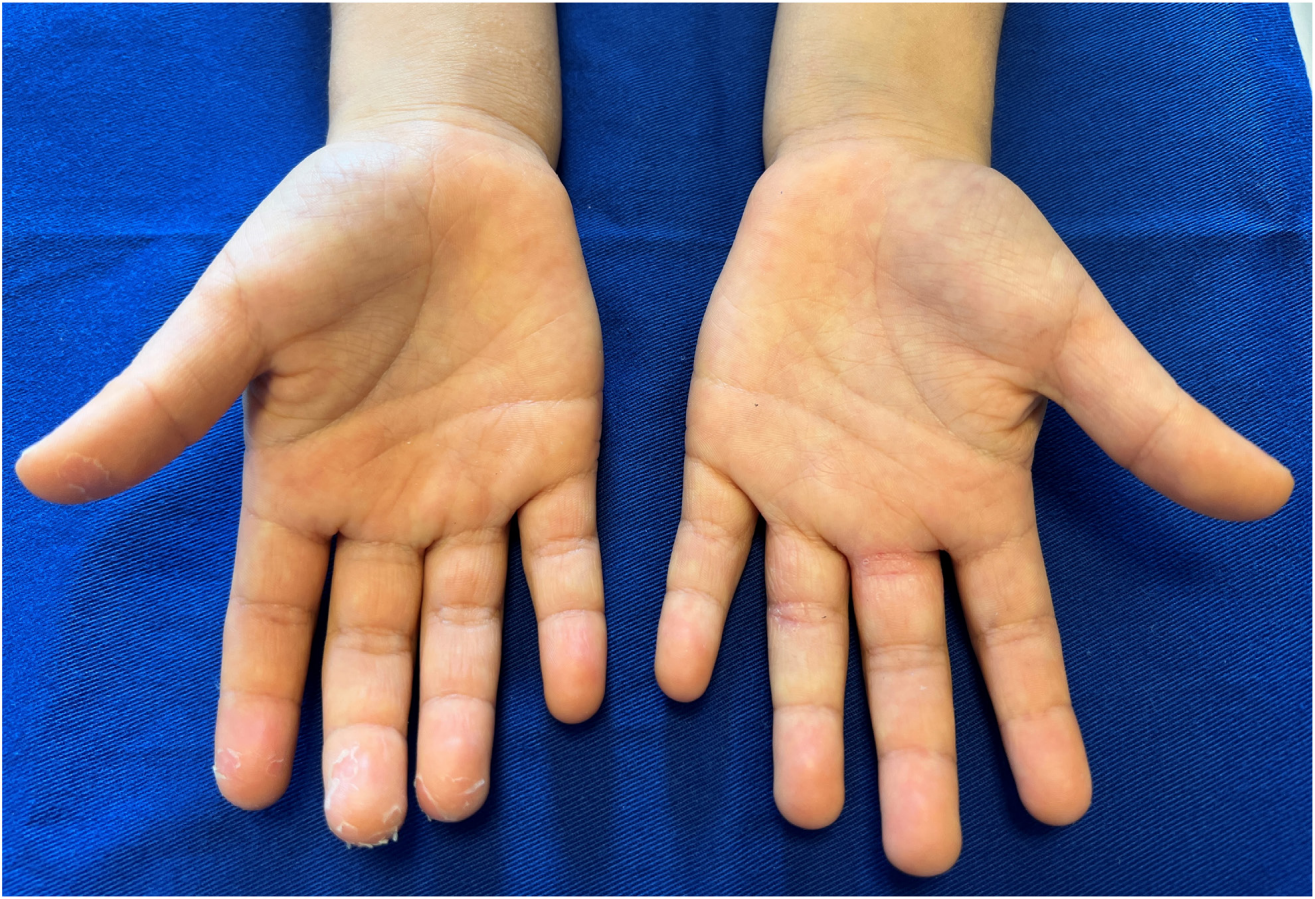
Findings of both hands. Membranous desquamation is visible on the first to fourth fingers of the right hand, with no erythema and no swelling on the palms or fingers. The fingers on the left hand show redness and desquamation due to atopic dermatitis and no membranous desquamation on the fingertips

**FIGURE 2:**
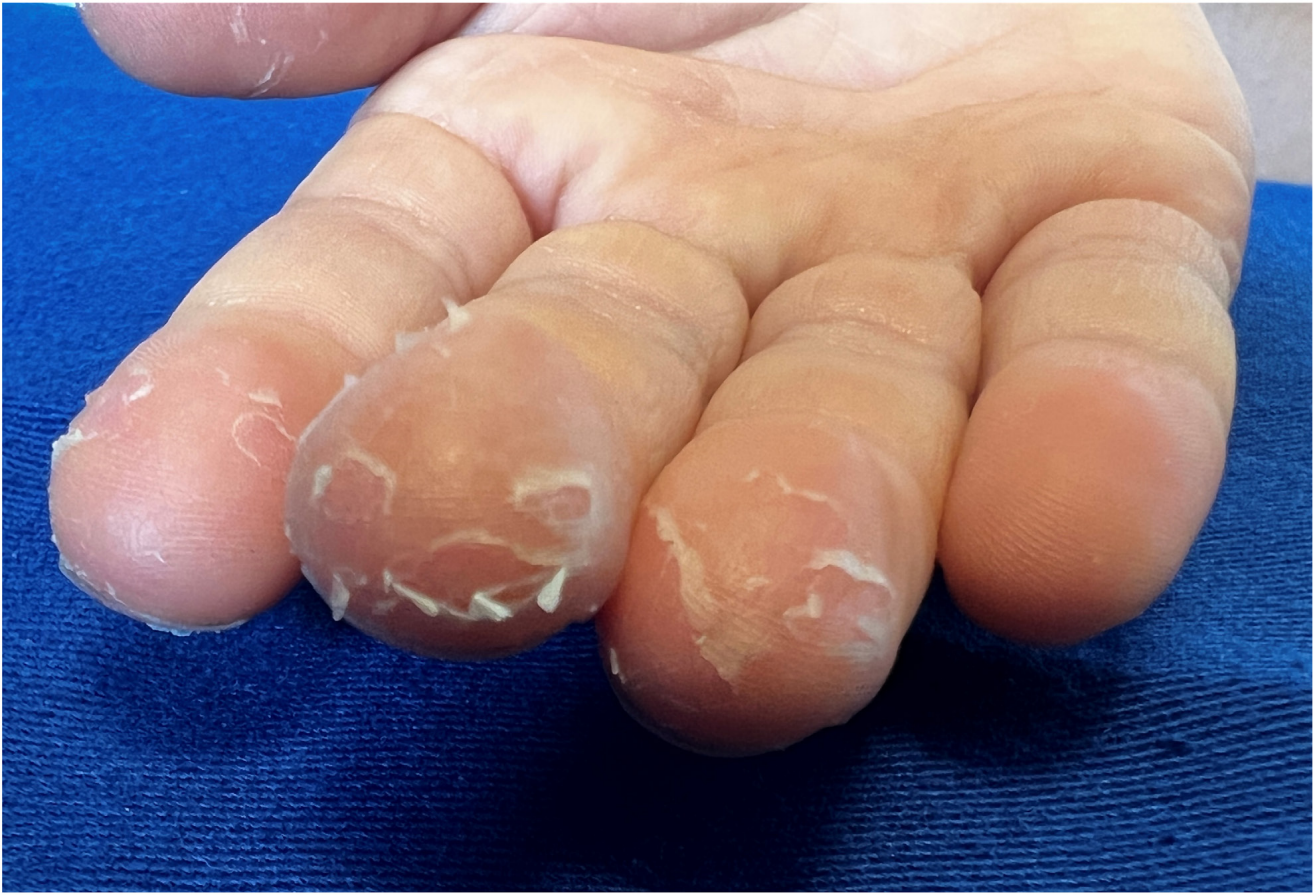
Magnified findings on the fingers of the right hand. Membranous desquamation is visible on the second to fourth fingers of the right hand

A 7‐year‐old Japanese boy with atopic dermatitis, who was fully vaccinated for coronavirus disease 2019 (COVID‐19), developed a wet cough with sputum on Day 0. His sister had had a positive result for COVID‐19 on a polymerase chain reaction test the previous day, so he was tested for and diagnosed with COVID‐19. He took 240 mg of acetaminophen for intermittent fever and headache, and the symptoms improved by Day 3. However, he developed membranous desquamation on the right hand on Day 4 (Figures 1 and 2), which improved without treatment. There was no erythema or swelling of the hand and no findings suggestive of Kawasaki disease after the onset of COVID‐19.

Most cases of membranous desquamation in COVID‐19 have been reported in severely affected pediatric patients with multisystem inflammatory syndrome in children (MIS‐C); mild cases have rarely been reported.^1^ Patients with Kawasaki disease typically present with desquamation of the skin around the nails, with erythema and edema.^2^ Therefore, differentiation from other diseases presenting with the same signs is essential for the earliest treatment. COVID‐19 can cause membranous desquamation regardless of severity. Accordingly, in pediatric patients, the general condition, vital signs, and other physical findings should be carefully confirmed to rule out complications of MIS‐C or Kawasaki disease, as the incidences of these conditions are reportedly increasing during the COVID‐19 pandemic.^3^

## AUTHOR CONTRIBUTIONS

MT: was involved in the clinical care of the patient, literature search, study conception, and drafting and revision of the manuscript. RH: was involved in the literature search and drafting of the manuscript.

## FUNDING INFORMATION

No funding was received for this article.

## CONFLICT OF INTEREST

The authors state that they have no conflict of interest.

## ETHICAL APPROVAL

This report conforms to the provisions of the Declaration of Helsinki in 1995 (as revised in Brazil 2013).

## CONSENT

Written informed consent to publish this report was obtained from the patient in accordance with the journal's patient consent policy.

## Data Availability

The data that support the findings of this study are available from the corresponding author upon reasonable request.
